# Supplementary material for: Resistance Genes, Plasmids, Multilocus Sequence Typing (MLST), and Phenotypic Resistance of Non-Typhoidal Salmonella (NTS) Isolated from Slaughtered Chickens in Burkina Faso
Source: Antibiotics (Basel). 2022 Jun 8;11(6):782. doi: 10.3390/antibiotics11060782 (PMC9219948; doi:10.3390/antibiotics11060782)
Supplement: Supplementary file 1 [file antibiotics-11-00782-s001.zip › antibiotics-1717064-supplementary.pdf]

Supplemental Table S1. Characteristics of Salmonella isolated from slaughtered chickens.

Sample	Serotype	Antimicrobial Resistances Genes[a]	Partial Antimicrobial Resistance Genes	Phenotypic Resistance Profile[b,c]	Plasmid Repliconsc	Partial Plasmid Replicons	Chromosamal Point Mutationc	Multi-Locus Sequence Type (MLST)
**S38**	Albany or Duesseldorf	*aac*(6')-Iaa; *aph*(3')-Ia; *tet*(A)		TET	IncI1-I (Alpha)		parC p.T57S	292
**S39**	Chester	aac(6')-Iaa		ND	ND		parC p.T57S	411
**S47**	Hato	aac(6')-Iaa		ND	IncFIB (H89-PhagePlasmid)		parC p.T57S	Unknown
**S52**	Chester	aac(6')-Iaa		ND	ND		parC p.T57S	411
**S53**	Hato	aac(6')-Iaa		ND	ND		parC p.T57S	3899
**S58**	Telelkebir	aac(6')-Iaa; fosA7		ND	IncFIB(S)		parC p.T57S	2386
**S59**	Typhimurium	aac(6')-Iaa; blaTEM-1B; mph(A)		AMP; AMPSUL (A/S2); PIP; TICCLA(TIM2)	Col440I, IncFIB(S), IncFII(S), IncFII(pCoo)		ND	313
**S60**	Telelkebir	aac(6')-Iaa; fosA7		ND	ND		parC p.T57S	5494
**S63**	Hato	aac(6')-Iaa		ND	ND		parC p.T57S	3899
**S64**	Agona	aac(6')-Iaa; fosA7		ND	ND		parC p.T57S	7876
**S65**	Derby	aac(6')-Iaa		ND	ND		parC p.T57S	7119
**S66**	I 1,3,19:f,g:1,5	aac(6')-Iaa		ND	ND		parC p.T57S	Unknown
**S67**	Chester	aac(6')-Iaa		ND	ND		parC p.T57S	411
**S69**	Kentucky	aac(6')-laa; aac(3)-Id; aadA7; aph(3'')-Ib; aph(6)-Id; dfrA15; sul1; tet(A)	qacE	GEN; TET; TRISUL(SXT)	ND		parC p.T57S	314
**S71**	Virchow	aac(6')-Iaa		ND	IncI1-I (Alpha)		ND	181

**S72**	Amoutive	aac(6')-Iaa		ND	ND		parC p.T57S	Unknown
**S74**	Kentucky	aac(6')-Iaa; aac(3)-Id; aadA7; aph(3'')-Ib; aph(6)-Id; dfrA15; sul1; tet(A)	qacE	GEN; TET; TRISUL(SXT)	ND		parC p.T57S	314
**S75**	Typhimurium	aac(6')-Iaa		GEN; TET; TRISUL(SXT)	ColRNAI, IncFIB(S), IncFII(S), IncX1		ND	19
**S80**	Derby	aac(6')-Iaa		ND	Col8282, IncFIB (H89-PhagePlasmid)	IncB/O/K/Z	parC p.T57S	5421
**S82**	Chester	aac(6')-Iaa		ND	ND		parC p.T57S	411
**S83**	Typhimurium	aac(6')-Iaa		ND	ColRNAI, IncFIB(S), IncFII(S), IncX1		ND	19
**S86**	Brancaster	aac(6')-Iaa		ND	ND		parC p.T57S	Unknown
**S90**	Gaminara	aac(6')-Iaa		ND	ND		parC p.T57S	2152
**S91**	Derby	aac(6')-Iaa		ND	ND		parC p.T57S	7882
**S92**	Derby	aac(6')-Iaa		ND	ND		parC p.T57S	7882
**S93**	Schwarzengrund	aac(6')-Iaa		ND	ND		parC p.T57S	96
**S94**	Derby	aac(6')-Iaa		ND	ND		parC p.T57S	7880
**S96**	Farmingdale	aac(6')-Iaa		ND	ND		parC p.T57S	Uknown
**S97**	Derby	aac(6')-Iaa		ND	ND		parC p.T57S	7880
**S99**	Anatum	aac(6')-Iaa		ND	ND		parC p.T57S	5197
**S102**	Bredeney	aac(6')-Iaa		ND	ND		parC p.T57S	306
**S104**	Alexanderplatz	aac(6')-Iaa		ND	IncFII(S)	IncB/O/K/Z	parC p.T57S	Unknown
**S105**	Derby	aac(6')-Iaa		ND	ND		parC p.T57S	7882
**S106**	Rechovot	aac(6')-Iaa		ND	ND		parC p.T57S	Unknown
**S107**	Bredeney	aac(6')-Iaa		ND	ND		parC p.T57S	306
**S109**	Derby	aac(6')-Iaa		ND	ND		parC p.T57S	7882
**S110**	Eastbourne	aac(6')-Iaa		ND	ND		parC p.T57S	414

**S112**	Hato	aac(6')-Iaa	ND	ND		parC p.T57S	3997
**S114**	Hato	aac(6')-Iaa	ND	ND		parC p.T57S	Unknown
**S115**	Derby	aac(6')-Iaa	ND	ND		parC p.T57S	7880
**S118**	Poona	aac(6')-Iaa	ND	ND		parC p.T57S	308
**S120**	Derby	aac(6')-Iaa	ND	ND		parC p.T57S	7882
**S121**	Poona	aac(6')-Iaa	ND	ND		parC p.T57S	308
**S123**	Bredeney	aac(6')-Iaa	ND	ND		parC p.T57S	306
**S124**	Derby	aac(6')-Iaa	ND	ND		parC p.T57S	7880
**S125**	Hato	aac(6')-Iaa	ND	ND		parC p.T57S	Unknown
**S126**	Rechovot	aac(6')-Iaa	ND	ND		parC p.T57S	Unknown
**S132**	Derby	aac(6')-Iaa	ND	ND		parC p.T57S	7882
**S133**	Derby	aac(6')-Iaa	ND	ND		parC p.T57S	7882
**S140**	Alexanderplatz	aac(6')-Iaa	ND	IncFII(S)	IncB/O/K/Z	parC p.T57S	Unknown
**S143**	Alexanderplatz	aac(6')-Iaa	ND	IncFII(S)	IncB/O/K/Z	parC p.T57S	Unknown
**S145**	Tennessee	aac(6')-Iaa	ND	ND		parC p.T57S	8398
**S147**	Drac	aac(6')-Iaa	ND	ND		parC p.T57S	2221
**S148**	Muenster	aac(6')-Iaa	ND	ND		parC p.T57S	321
**S149**	Muenster	aac(6')-Iaa	ND	ND		parC p.T57S	321
**S150**	Muenster	aac(6')-Iaa	ND	ND		parC p.T57S	321
**S151**	Muenster	aac(6')-Iaa	ND	ND		parC p.T57S	321
**S152**	Muenster	aac(6')-Iaa	ND	ND		parC p.T57S	321
**S153**	Muenster	aac(6')-Iaa	ND	ND		parC p.T57S	321
**S154**	Muenster	aac(6')-Iaa	ND	ND		parC p.T57S	321
**S155**	Poona	aac(6')-Iaa	ND	ND		parC p.T57S	608
**S156**	Poona	aac(6')-Iaa	ND	ND		parC p.T57S	608
**S162**	I 1,3,19:b:-	aac(6')-Iaa	ND	ND		parC p.T57S	Unknown
**S163**	Derby	aac(6')-Iaa	ND	IncFIB (H89-PhagePlasmid)		parC p.T57S	3135
**S164**	I 1,3,19:b:-	aac(6')-Iaa	ND	ND		parC p.T57S	Unknown

**S165**	Derby	aac(6')-Iaa		ND	IncFIB (H89-PhagePlasmid)		parC p.T57S	3135
**S167**	I 1,3,19:b:-	aac(6')-Iaa		ND	ND		parC p.T57S	Unknown
**S168**	Typhimurium	aac(6')-Iaa		ND	ColRNAI, IncFIB(S), IncFII(S), IncX1		ND	19
**S169**	Hato	aac(6')-Iaa		ND	IncFIB (H89-PhagePlasmid)		parC p.T57S	3292
**S170**	Derby	aac(6')-Iaa		ND	IncFIB (H89-PhagePlasmid)		parC p.T57S	3135
**S171**	Derby	aac(6')-Iaa; fosA7		ND	Col(pHAD28), IncI1-I (Alpha)		parC p.T57S	7881
**S172**	Kentucky	aac(6')-Iaa; dfrA15; sul1	qacE	TRISUL(SXT)	ND		parC p.T57S	314
**S175**	Hato	aac(6')-Iaa; aadA1; sul2; tet(A)	dfrA14; aph(3")-Ib; aph(6)-Id; aph(3')-Ia	TET; TRISUL(SXT)	IncI1-I (Alpha)	IncQ1	parC p.T57S	3899
**S183**	Derby	aac(6')-Iaa; aph(3")-Ib; aph(6)-Id; sul2; tet(A)		MIN; TET	IncFIB (H89-PhagePlasmid), IncQ1		parC p.T57S	3135
**S184**	Hato	aac(6')-Iaa; aph(3')-Ia; [aph(3")-Ib]; aph(6)-Id; dfrA14; sul2		TRISUL(SXT)	IncI1-I (Alpha)	IncQ1	parC p.T57S	3899
**S185**	Hato	aac(6')-Iaa; aadA1; aph(3')-Ia; dfrA14; sul2; tet(A)	aph(3")-Ib; aph(6)-Id	MIN; TET; TRISUL(SXT)	IncI1-I (Alpha)	IncQ1	parC p.T57S	3899
**S186**	Hato	aac(6')-Iaa; aadA1; sul2; tet(A)	dfrA14; aph(3")-Ib; aph(6)-Id; aph(3')-Ia	TET; TRISUL(SXT)	IncI1-I (Alpha)	IncQ1	parC p.T57S	3899

**S187**	Hato	aac(6')-Iaa; aadA1; aph(3')-Ia; aph(6)-Id; sul2; tet(A)	dfrA14; aph(3")-Ib	TET; TRISUL(SXT)	IncI1-I (Alpha)	IncQ1	parC p.T57S	3899
**S188**	Hato	aac(6')-Iaa; aadA1; aph(3')-Ia; [aph(3")-Ib]; sul2; tet(A)	dfrA14; aph(6)-Id	MIN; TET; TRISUL(SXT)	IncI1-I (Alpha)	IncQ1	parC p.T57S	3899
**S191**	Hato	aac(6')-Iaa; aadA1; aph(3')-Ia; aph(6)-Id; dfrA14; sul2; tet(A)	aph(3")-Ib	MIN; TET; TRISUL(SXT)	IncI1-I (Alpha)	IncQ1	parC p.T57S	3899
**S194**	Hato	aac(6')-Iaa; aadA1; aph(3')-Ia; [aph(3")-Ib]; sul2; tet(A)	dfrA14; aph(6)-Id	TET; TRISUL(SXT)	IncI1-I (Alpha)	IncQ1	parC p.T57S	3899
**S196**	Hato	aac(6')-Iaa; aadA1; aph(3')-Ia; [aph(3")-Ib]; sul2; tet(A)	dfrA14; aph(6)-Id	TET; TRISUL(SXT)	IncI1-I (Alpha)	IncQ1	parC p.T57S	3899
**S197**	Hato	aac(6')-Iaa; aadA1; sul2; tet(A)	dfrA14; aph(3")-Ib; aph(6)-Id	TET; TRISUL(SXT)	IncI1-I (Alpha)	IncQ1	parC p.T57S	3899
**S198**	Derby	aac(6')-Iaa; aph(3")-Ib; aph(6)-Id; sul2; tet(A)		MIN; TET	IncQ1		parC p.T57S	3135
**S199**	Derby	aac(6')-Iaa; aph(3")-Ib; aph(6)-Id; sul2; tet(A)		MIN; TET	Col(pHAD28), Col8282, IncFIB (H89-PhagePlasmid), IncQ1		parC p.T57S	3135
**S200**	Hato	aac(6')-Iaa; aadA1; aph(3')-Ia; aph(6)-Id; dfrA14; sul2; tet(A)		TET; TRISUL(SXT)	IncI1-I (Alpha)	IncQ1	parC p.T57S	3899

**S201**	Hato	aac(6')-Iaa; aadA1; aph(3')-Ia; [aph(3'')-Ib]; aph(6)-Id; dfrA14; sul2; tet(A)		TRISUL(SXT)	IncI1-I (Alpha)	IncQ1	parC p.T57S	3899
**S202**	Hato	aac(6')-Iaa; aadA1; aph(3')-Ia; [aph(3'')-Ib]; aph(6)-Id; dfrA14; sul2; tet(A)		TET; TRISUL(SXT)	IncI1-I (Alpha)	IncQ1	parC p.T57S	3899
**S203**	Hato	aac(6')-Iaa; aadA1; aph(3')-Ia; sul2; tet(A)	dfrA14; aph(3'')-Ib; aph(6)-Id	TET; TRISUL(SXT)	IncI1-I (Alpha)	IncQ1	parC p.T57S	3899
**S204**	Hato	aac(6')-Iaa; aadA1; aph(3')-Ia; aph(6)-Id; sul2; tet(A)	dfrA14; aph(3'')-Ib	TET; TRISUL(SXT)	IncI1-I (Alpha)	IncQ1	parC p.T57S	3899
**S207**	Hato	aac(6')-Iaa; aadA1; aph(3')-Ia; [aph(3'')-Ib]; aph(6)-Id; dfrA14; sul2; tet(A)		TET; TRISUL(SXT)	IncI1-I (Alpha)	IncQ1	parC p.T57S	3899
**S208**	Hato	aac(6')-Iaa; aadA1; aph(3')-Ia; sul2; tet(A)	dfrA14; aph(3'')-Ib; aph(6)-Id	TET; TRISUL(SXT)	IncI1-I (Alpha)	IncQ1	parC p.T57S	3899
**S209**	Hato	aac(6')-Iaa; aadA1; aph(3')-Ia; sul2; tet(A)	dfrA14; aph(3'')-Ib; aph(6)-Id	TET; TRISUL(SXT)	IncI1-I (Alpha)	IncQ1	parC p.T57S	3899
**S212**	Hato	aac(6')-Iaa; aph(3'')-Ib; aph(6)-Id; sul2		ND	IncI1-I (Alpha)	IncQ1	parC p.T57S	3899
**S216**	Hato	aac(6')-Iaa; aadA1; aph(3')-Ia; sul2; tet(A)	dfrA14; aph(3'')-Ib; aph(6)-Id	TET; TRISUL(SXT)	IncI1-I (Alpha)	IncQ1	parC p.T57S	3899

**S219**	Hato	aac(6')-Iaa; aadA1; aph(3')-Ia; [aph(3'')-Ib]; sul2; tet(A)	dfrA14; aph(6)-Id	TET; TRISUL(SXT)	IncI1-I (Alpha)	IncQ1	parC p.T57S	3899
**S248**	Derby	aac(6')-Iaa; aph(3'')-Ib; aph(6)-Id; sul2; tet(A)		TET	Col8282, IncFIB (H89-PhagePlasmid), IncQ1	IncB/O/K/Z	parC p.T57S	Unknown
**S249**	Derby	aac(6')-Iaa; aph(3'')-Ib; aph(6)-Id; sul2; tet(A)		TET	Col8282, IncFIB (H89-PhagePlasmid), IncQ1	IncB/O/K/Z	parC p.T57S	Unknown
**S251**	Derby	aac(6')-Iaa; aph(3'')-Ib; aph(6)-Id; fosA7; sul2; tet(A)		MIN; TET	Col(pHAD28), IncI1-I (Alpha)		parC p.T57S	7881
**S252**	Tennessee	aac(6')-Iaa		ND	ND		parC p.T57S	8398
**S253**	I 1,3,19:b:-	aac(6')-Iaa		ND	ND		parC p.T57S	Unknown
**S255**	Kentucky	aac(6')-Iaa; aac(3)-Id; aadA7; aph(3'')-Ib; aph(6)-Id; dfrA15; sul1; tet(A)	qacE	GEN; TET; TRISUL(SXT)	ND		parC p.T57S	314

[a] Genes in brackets [ ] are complete but disrupted by an insertion

[b] Abbreviations indicate: TICCLA (TIM2), Ticarcillin/clavulanic acid; TET, Tetracycline; MIN, Minocycline; TRISUL(SXT), Trimethoprim/Sulfamethoxazole; PIP, Piperacillin; GEN, Gentamicin, AMPSUL (A/S2), Ampicillin/sulbactam; AMP, Ampicillin.

[c] ND indicates “not detected”
